# *Mycoplasma hominis *necrotizing pleuropneumonia in a previously healthy adolescent

**DOI:** 10.1186/1471-2334-10-335

**Published:** 2010-11-24

**Authors:** Andres Pascual, Marie-Helene Perez, Katia Jaton, Gaudenz Hafen, Stefano Di Bernardo, Jacques Cotting, Gilbert Greub, Bernard Vaudaux

**Affiliations:** 1 Infectious Diseases Service, Department of Medicine, Centre Hospitalier Universitaire Vaudois and University of Lausanne, 1011, Lausanne, Switzerland; 2 Pediatric Intensive Care Unit, Centre Hospitalier Universitaire Vaudois and University of Lausanne, 1011, Lausanne, Switzerland; 3 Pediatric Service, Department of Pediatrics, Centre Hospitalier Universitaire Vaudois and University of Lausanne, 1011, Lausanne, Switzerland; 4 Institute of Microbiology, Centre Hospitalier Universitaire Vaudois and University of Lausanne, 1011, Lausanne, Switzerland

## Abstract

**Background:**

*Mycoplasma hominis *is a fastidious micro-organism causing systemic infections in the neonate and genital infections in the adult. It can also be the cause of serious extra-genital infections, mainly in immunosuppressed or predisposed subjects.

**Case Presentation:**

We describe a case of severe pneumonia and pericarditis due to *Mycoplasma hominis *in a previously healthy adolescent who did not respond to initial therapy.

**Conclusions:**

*Mycoplasma hominis *could be an underestimated cause of severe pneumonia in immunocompetent patients and should be particularly suspected in those not responding to standard therapy.

## Background

*Mycoplasma hominis (M. hominis*) belongs to the order of Mycoplasmatales, prokaryotes that lack a cell wall. This characteristic has implications for antibiotic therapy as many commonly used antibiotics act by inhibiting the bacterial cell wall synthesis. Mycoplasma organisms have fastidious growth requirements and are often difficult to culture on a cell-free medium. A recent analysis of the *M. hominis *genome sequence revealed that it is the second smallest genome among self-replicating free living organisms with a predicted genome of 537 coding genes [1]. *M. hominis *is an opportunistic organism that is uncommonly associated with infection in humans. However, it is frequently isolated from the urogenital and respiratory tract of asymptomatic healthy persons [2]. Infants may be colonized following vaginal delivery but *M. hominis *is rarely recovered from prepubertal children. Colonization in postpubertal individuals results mainly from sexual contact [3].

*M. hominis *exceptionally penetrates the submucosal layer, unless the patient is immunosupressed or traumatized (through accident or instrumentation). In this case, it can invade the bloodstream and disseminate to many different organs and tissues throughout the body, causing extragenital infections [4]. However, extragenital infections are uncommon in healthy patients and cases of pneumonia are exceptional.

*M. hominis *is an organism recognized to be intrinsically resistant to macrolides. In addition, there is recent evidence of increasing resistance to tetracycline and emerging resistance to fluoroquinolones [5-7].

## Case Presentation

A previously healthy 15-year-old sexually active female came to the emergency room because of dyspnoea, fever and right thoracic pain. The physical examination was suggestive of right sided pneumonia and laboratory tests revealed a marked leukocytosis (39'800/mm^3 ^WBC, 89% neutrophils; 74% segmented and 15% bands) and an elevated C-reactive protein level (>250 mg/L). The chest X-ray confirmed the clinical suspicion of right lobar pneumonia and an outpatient treatment of oral clarithromycin (500 mg bid) was started. The absence of clinical improvement and the onset of a pleural effusion on the fourth day of antibiotic therapy lead to admission to our hospital and the addition of i.v. ceftriaxone (2 g bid) to the macrolide regimen.

As the high fever persisted, a chest CT scan was done on the seventh day of antibiotic treatment, showing a bilateral necrotizing pneumonia as well as a bilateral pleural effusion. At this time, on the basis of a presumed polymicrobial infection including anaerobes, a treatment of i.v. amoxicillin/clavulanate (2.2 g tid) was substituted to the previous clarithromycin-ceftriaxone regimen.

Two days later the patient was transferred to the paediatric intensive care unit because of rapid worsening of her symptoms with the risk of respiratory failure. Amoxicillin/clavulanate therapy was switched to imipenem-cilastatin (500 mg i.v. qid) and vancomycin (750 mg i.v. tid). Cultures of pleural effusion for aerobic and anaerobic pathogens obtained by needle aspiration were negative. *M. hominis *was then detected by eubacterial PCR [DNA extracts were amplified using PCR targeting the 16S rRNA as follows: forward primer (Bak11wF): 5'-AGTTTGATCMTGGCTCAG-3', reverse Primer (Pc3 mod): 5'-GGACTACHAGGGTATCTAAT-3', sequencing primer: Bak11wf and Bak533 r: 5'-TTACCGCGGCTGCTGGCAC-] [3], and confirmed by a *M. hominis *specific Taqman PCR (98,500,000 copies/mL). The primers of the TaqMan PCR used were: Forward primer: 5'-TTTGGTCAAGTCCTGCAACGA-3', Reverse Primer: 5'-CCCCACCTTCCTCCCAGTTA-3' and probe: FAM-TTACTAACATTAAGTTGAGGACTCTA-MGB, targeting the *M. hominis 16 rRNA *gene sequence [8], and further confirmed by specific culture (Mycoplasma IST, bioMérieux, France).

The antibiotic susceptibility tests revealed resistance to macrolides and susceptibility to tetracyclines and quinolones, leading to the addition of doxycycline (100 mg bid) twelve days after the start of symptoms.

**Figure 1:**
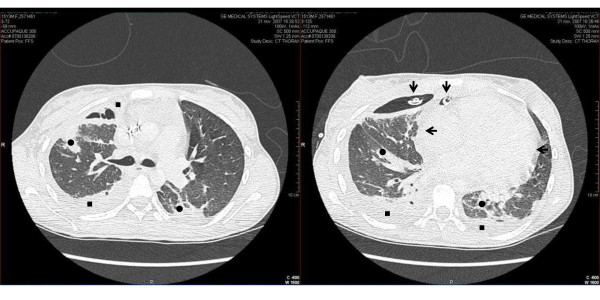
**CT scan of the thorax showing bilateral necrotizing pneumonia (black circles) and pleural effusion (black squares) as well as pericardial effusion (leftwards arrows)**. Note the presence of a chest tube inserted into the pleural cavity (in communication with the lung abscess) and a drainage tube into the pericardial cavity (downwards arrows).

A new CT scan showed a larger amount of pleural fluid as well as a pericardial effusion (Figure 1).

Because of worsening respiratory and haemodynamic conditions, the pericardial and pleural effusions were evacuated by pericadiectomy with window and pleural drainage by chest tube insertion. Any respiratory support (other than oxygen supplementation) was needed thereafter. *M. hominis *DNA was also amplified in the pericardial fluid by specific PCR (85 copies/mL). Standard axenic cultures and specific PCR assays for *Streptococcus pneumoniae *and *Staphylococcus aureus*, as well as *Legionella pneumophila *urinary antigen test were negative in all specimens. Complement-fixing antibody to *Mycoplasma pneumoniae *showed no increase in titers.

Imipenem-cilastin and vancomycine were discontinued while doxycycline was administered for a total of 2 weeks. This treatment was associated with clinical and biological improvement and the patient could be discharged from the hospital on day 19. At this time, the CRP level was down to 16 mg/L. Finally the patient completely recovered without any sequel on the chest X-ray.

## Discussion

We described a case of severe pneumonia and pericarditis due to *M. hominis *in a previously healthy adolescent who completely recovered under doxycycline therapy. *M. hominis *was detected from the pleural and pericardial fluids by eubacterial PCR assay and subsequent specific PCR assay and culture. No alternative pathogen has been identified by culture, molecular or serological diagnostic procedures.

*M. hominis *may cause genital infections in adults and may be involved in neonatal infections. Serious extra-genital infections, such as brain abscess, pneumonia, mediastinitis, pericarditis, endocarditis, osteitis and arthritis, wound infections, peritonitis, and pyelonephritis have been reported, mainly in immunosuppressed patients [9-12]. However, such cases have also been described in immunocompetent patients, particularly in individuals with predisposing factors such as trauma, altered cardiorespiratory function and complicated urogenital manipulations or surgery.

Our review of the literature identified 11 cases of *M. hominis *pneumonia in immunocompetent patients (Additional file 1) [13-17].

Six cases occurred in patients with pre-existing co-morbidities (trauma or surgery, subarachnoid haemorrhage, and oesophageal carcinoma), one case in a pregnant woman, and four cases in patients with no known predisposing conditions.

Complete resolution has been described in the five patients who received antibiotic therapy active against *M. hominis*. In contrast, among those receiving an inadequate antibiotic therapy, four died and two recovered suggesting that untreated *M. hominis *infection may aggravates pre-existing conditions and lead to death.

Initially, our patient experienced a severe worsening of her clinical condition while on antibiotics active against common respiratory pathogens, but has fully recovered after receiving an antimicrobial drug active against *M. hominis*.

*M. hominis *may be an underestimated cause of severe pneumonia in previously healthy patients, particularly when other common etiological agents have been ruled out in those not responding to standard therapy.

The *M. hominis *strain isolated in our patient was susceptible to both tetracyclines and quinolones. We used a susceptibility assay based on breakpoint cut offs set according to the recommendations of the Clinical and Laboratory Standards Institute (CLSI) [18].

Unpublished data from our center on the antibiotic susceptibility of *M. hominis *strains isolated from the genital and respiratory tracts between 2000 and 2008 are in accordance with the data from the literature: 55% are susceptible to ciprofloxacin (5/9), 100% are susceptible to doxycycline (11/11), and 100% are resistant to clarithromycin (9/9).

*M. hominis *is intrinsically resistant to macrolides [19-21], and tetracyclines have been considered the drugs of choice. However, the therapeutic activity of tetracyclines may become unreliable due to resistance phenomena induced by previous antibiotic exposure. Moreover, they are no longer a valid therapeutic option in some areas [5,7]. As no resistance to levofloxacin (or other newer fluoroquinolones) and clindamycin is yet identified, these drugs could be a suitable therapeutic alternative [5,7,20,22,23]. The increasing resistance of *M. hominis *strains to antibiotics makes guidance of therapy by in vitro susceptibility tests of paramount importance in invasive infections leading to life-threatening situations.

## Conclusions

This case report shows the role of *M. hominis *as a possible etiologic agent in a previous healthy adolescent suffering a severe and complicated pneumonia not responding to macrolide and β-lactam agents.

The eubacterial PCR is a useful tool to detect unusual pathogens.

The efficacy of traditional drugs such as tetracyclines on *M. hominis *is affected by increasing resistance, making susceptibility testing an important issue nowadays. The newest fluoroquinolones are an attractive option to treat invasive infections caused by *M. hominis*.

## Consent

Written informed consent was obtained from the patient and her mother for publication of this case report. A copy of the written consent is available for review by the Editor-in-Chief of this journal.

## Competing interests

The authors declare that they have no competing interests.

## Authors' contributions

JC, MHP, GH, SDB, BV and AP: have been involved in patient clinical care, and in acquisition and interpretation of data. MHP, AP and BV have been involved in drafting the manuscript. GG and KJ carried out the standard and specific microbiologic tests and the molecular genetic studies. GH, GG, MHP, AP and BV: have reviewed the manuscript. All authors read and approved the final manuscript.

## Pre-publication history

The pre-publication history for this paper can be accessed here:

http://www.biomedcentral.com/1471-2334/10/335/prepub

## Supplementary Material

Additional file 1 Table S1: Summary of previously reported *Mycoplasma hominis *pneumonia in immunocompetent patients. Click here for file
