# Supplementary material for: Mycoplasma hominis necrotizing pleuropneumonia in a previously healthy adolescent
Source: BMC Infect Dis. 2010 Nov 24;10:335. doi: 10.1186/1471-2334-10-335 (PMC3006422; doi:10.1186/1471-2334-10-335)
Supplement: Additional file 1 — Table S1: Summary of previously reported Mycoplasma hominis pneumonia in immunocompetent patients. [file 1471-2334-10-335-S1.PDF]

Table 1. Summary of previously reported *Mycoplasma hominis* pneumonia in immunocompetent patients

**Patient no.**	Age (years)	Isolated Pathogens	Site of isolation	Reason for Hospital admission	Risk factor, prior illness	Initial Empirical treatment	Adjusted therapy	Outcome	Reference
**1**	24	*M. hominis*	Blood, Ankle Wound, Pleural Fluid	Complicated pneumonia	Polytrauma	Erythromycin	-	Resolved	[17]
**2**	21	*M. hominis and Klebsiella spp*	Pleural Fluid	Complicated pneumonia	No risk factors known	Cefazolin, cefoxitin, penicillin and erythromycin	-	Resolved	[15]
**3**	32	*M. hominis*	Pleural Fluid	Complicated pneumonia	Pregnancy	Netilmycin, ceftriaxone and erythromycin	Doxycycline	Resolved	[13]
**4**	35	*M. hominis*	Blood	Pneumonia	No risk factors known	Cefotaxime, penicillin, erythromycin and gentamicin	-	Died	[16]
**5**	19	*M. hominis*	Tracheal aspirate	Pneumonia	Head trauma	Rolitetracycline	-	Resolved	[16]
**6**	39	*M. hominis*	Tracheal aspirate	Pneumonia	Subdural hematoma	Ceftriaxone and erythromycin	-	Died	[16]
**7**	24	*M. hominis*	Lung autopsy	Pneumonia	No risk factors known	Ceftriaxone, erythromycin and gentamicin	-	Died	[16]
**8**	25	*M. hominis*	BAL	Multiple fractures	No risk factors known	Piperacillin/tazobactam	Ciprofloxacin	Resolved	[14]
**9**	46	*M. hominis*	BAL	Hypertension alcoholism	Subarachnoid hemorrhage	Piperacillin/tazobactam	Levofloxacin	Resolved	[14]
**10**	42	*M. hominis and P.aeruginosa*	BAL	Esophageal carcinoma tracheoeso- phageal fistula	Septic shock	Imipenem	-	Died	[14]
**11**	36	*M. hominis*	BAL	Hepatitis C	Polytrauma	Cefepime and levofloxacin	Levofloxacin	Resolved	[14]

BAL: Bronchoalveolar lavage
